# Differences in health-related quality of life between HIV-positive and HIV-negative people in Zambia and South Africa: a cross-sectional baseline survey of the HPTN 071 (PopART) trial

**DOI:** 10.1016/S2214-109X(17)30367-4

**Published:** 2017-09-27

**Authors:** Ranjeeta Thomas, Ronelle Burger, Abigail Harper, Sarah Kanema, Lawrence Mwenge, Nosivuyile Vanqa, Nomtha Bell-Mandla, Peter C Smith, Sian Floyd, Peter Bock, Helen Ayles, Nulda Beyers, Deborah Donnell, Sarah Fidler, Richard Hayes, Katharina Hauck, HPTN 071 (PopART) Study Team, James Hargreaves, Deborah Watson-Jones, Peter Godfrey-Faussett, Anne Cori, Mike Pickles, Nomtha Mandla, Blia Yang, Anelet James, Redwaan Vermaak, Nozizwe Makola, Graeme Hoddinott, Vikesh Naidoo, Virginia Bond, Musonda Simwinga, Alwyn Mwinga, Barry Kosloff, Mohammed Limbada, Justin Bwalya, Chepela Ngulube, Christophe Fraser, Susan Eshleman, Yaw Agyei, Vanessa Cummings, Denni Catalano, Lynda Emel, Lisa Bunts, Heather Noble, David Burns, Alain Kouda, Niru Sista, Ayana Moore, Rhonda White, Tanette Headen, Eric Miller, Kathy Hinson, Sten Vermund, Mark Barnes, Lyn Horn, Albert Mwango, Megan Baldwin, Shauna Wolf, Erin Hughes

**Affiliations:** a Department of Economics, Stellenbosch University, Stellenbosch, South Africa; b Desmond Tutu TB Centre, Department of Paediatrics and Child Health, Stellenbosch University, Cape Town, South Africa; c ZAMBART Project, Ridgeway Campus, University of Zambia, Lusaka, Zambia; d Imperial College Business School, Imperial College London, London, UK; e Department of Medicine, Imperial College London, London, UK; f Department of Infectious Disease Epidemiology, Imperial College London, London, UK; g Department of Infectious Disease Epidemiology, Faculty of Epidemiology and Population Health, London School of Hygiene & Tropical Medicine, London, UK; h Department of Clinical Research, Faculty of Infectious and Tropical Diseases, London School of Hygiene & Tropical Medicine, London, UK; i Vaccine and Infectious Disease Division, Fred Hutchinson Cancer Research Center, Seattle, WA, USA

## Abstract

**Background:**

The life expectancy of HIV-positive individuals receiving antiretroviral therapy (ART) is approaching that of HIV-negative people. However, little is known about how these populations compare in terms of health-related quality of life (HRQoL). We aimed to compare HRQoL between HIV-positive and HIV-negative people in Zambia and South Africa.

**Methods:**

As part of the HPTN 071 (PopART) study, data from adults aged 18–44 years were gathered between Nov 28, 2013, and March 31, 2015, in large cross-sectional surveys of random samples of the general population in 21 communities in Zambia and South Africa. HRQoL data were collected with a standardised generic measure of health across five domains. We used β-distributed multivariable models to analyse differences in HRQoL scores between HIV-negative and HIV-positive individuals who were unaware of their status; aware, but not in HIV care; in HIV care, but who had not initiated ART; on ART for less than 5 years; and on ART for 5 years or more. We included controls for sociodemographic variables, herpes simplex virus type-2 status, and recreational drug use.

**Findings:**

We obtained data for 19 750 respondents in Zambia and 18 941 respondents in South Africa. Laboratory-confirmed HIV status was available for 19 330 respondents in Zambia and 18 004 respondents in South Africa; 4128 (21%) of these 19 330 respondents in Zambia and 4012 (22%) of 18 004 respondents in South Africa had laboratory-confirmed HIV. We obtained complete HRQoL information for 19 637 respondents in Zambia and 18 429 respondents in South Africa. HRQoL scores did not differ significantly between individuals who had initiated ART more than 5 years previously and HIV-negative individuals, neither in Zambia (change in mean score −0·002, 95% CI −0·01 to 0·001; p=0·219) nor in South Africa (0·000, −0·002 to 0·003; p=0·939). However, scores did differ between HIV-positive individuals who had initiated ART less than 5 years previously and HIV-negative individuals in Zambia (−0·006, 95% CI −0·008 to −0·003; p<0·0001). A large proportion of people with clinically confirmed HIV were unaware of being HIV-positive (1768 [43%] of 4128 people in Zambia and 2026 [50%] of 4012 people in South Africa) and reported good HRQoL, with no significant differences from that of HIV-negative people (change in mean HRQoL score −0·001, 95% CI −0·003 to 0·001, p=0·216; and 0·001, −0·001 to 0·001, p=0·997, respectively). In South Africa, HRQoL scores were lower in HIV-positive individuals who were aware of their status but not enrolled in HIV care (change in mean HRQoL −0·004, 95% CI −0·01 to −0·001; p=0·010) and those in HIV care but not on ART (−0·008, −0·01 to −0·004; p=0·001) than in HIV-negative people, but the magnitudes of difference were small.

**Interpretation:**

ART is successful in helping to reduce inequalities in HRQoL between HIV-positive and HIV-negative individuals in this general population sample. These findings highlight the importance of improving awareness of HIV status and expanding ART to prevent losses in HRQoL that occur with untreated HIV progression. The gains in HRQoL after individuals initiate ART could be substantial when scaled up to the population level.

**Funding:**

National Institute of Allergy and Infectious Diseases, National Institute on Drug Abuse, National Institute of Mental Health, President's Emergency Plan for AIDS Relief, International Initiative for Impact Evaluation, the Bill & Melinda Gates Foundation.

## Introduction

The 2015 UNAIDS Fast-Track targets are a call to action to protect the health of the roughly 19·8 million people globally with no access to antiretroviral therapy (ART). The targets stipulate that by 2020, 90% of people with HIV know their status, 90% of people who know their status are on ART, and 90% of people on ART have suppressed viral loads. However, to reach these ambitious targets, UNAIDS estimates that domestic and international investment in HIV programmes in low-income and middle-income countries (LMICs) will need to increase by about a third, from an estimated US$19·2 billion available in 2014, to $26·2 billion by 2020.^1^ It is difficult for policy makers to justify the large investments needed to step up HIV interventions from current health budgets when faced with many other urgent public health priorities.

**Research in context**

**Evidence before this study**

We searched MEDLINE, PubMed, and Embase on Feb 9, 2016, for studies published between Jan 1, 1995, and Dec 31, 2015, published in English, that compared the health-related quality of life (HRQoL) of people living with HIV with that of the general population across all World Bank income groups. We used the search terms “HIV”, “AIDS”, “quality-of-life”, and “population”. We excluded studies that focused exclusively on the health of HIV-positive individuals without comparison with the health of HIV-negative individuals or the general population, and studies that evaluated a specific health aspect (eg, depression) and not quality of life across all dimensions, that focused on specific populations (eg, pregnant mothers, diamond miners), or patients with adverse events, particular comorbidities, or co-infections. We identified five studies: three from high-income countries and two from South Africa. One study was published in 2014, and the others were at least 12 years old (one was from 2004, two from 2000, and one from 1996). HIV-positive patient populations differed between studies; two studies comprised 2864 and 3258 patients at all stages of disease, two studies focused on 72 and 134 patients at earlier disease stages (exclusion criterion CD4 cell count <200 per μL or acute or terminal illness), and one study focused on 123 patients with advanced disease (exclusion criterion CD4 cell count >200 per μL). All studies found that HRQoL was lower in HIV-positive individuals than in the general population. The two studies from South Africa found that HRQoL was compromised across all dimensions. The three studies from high-income countries found that HRQoL was most affected by emotional functioning. One study found that physical functioning was worse for patients with AIDS, but not for patients with asymptomatic disease. Almost all previous studies evaluated HRQoL in HIV patients who attended a clinic, participated in a clinical study, or were receiving health care. Because these individuals sought care, their health could have been compromised and they were therefore not representative of the general HIV-positive population.

**Added value of this study**

This study is one of the most extensive and robust analyses of differences in HRQoL among HIV-positive and HIV-negative individuals in a random sample of the general population in sub-Saharan Africa since the rapid scale up of antiretroviral therapy (ART). HIV status was determined from blood samples taken during the survey and confirmed with laboratory testing. We did a direct comparison of HRQoL between HIV-positive people and HIV-negative people. Furthermore, our study design enabled adjustment for confounders that were collected for both groups in the same way. The data are a random sample of the general population, thus giving an estimate of the HRQoL of all people living with HIV, not just the most ill. The study provides a rare insight into the HRQoL of HIV-positive individuals at different stages of engagement with HIV care, even those who were not aware of their status or who were aware but not in HIV care.

**Implications of all the available evidence**

Our results can be used to estimate how many quality-adjusted life-years could be gained with HIV treatment because of reductions in morbidity. This is crucial information for policy makers to comprehensively assess the societal worth of HIV interventions aimed at increasing the number of individuals receiving treatment.

A potentially large immediate benefit of ART, which has received little attention in policy debates, is its success in restoring the health-related quality of life (HRQoL) of people living with HIV. Studies of clinical cohorts have shown that most individuals at advanced stages of disease have improved health outcomes when on ART.^2^, ^3^ However, little evidence exists about the HRQoL of HIV-positive people at various stages of engagement in HIV care, when benchmarked against the attainable HRQoL of the HIV-negative population. Evidence about the effectiveness of ART in reducing the extreme inequalities in population health caused by HIV in high-burden settings is a crucial piece of evidence missing from the current debate. Such evidence would garner support for reducing the funding gap required to achieve the UNAIDS 2020 Fast-Track 90-90-90 targets.

We did this study to compare the HRQoL of people living with HIV with that of individuals not infected with HIV.

## Methods

### Study population and data

We analysed data from a large cross-sectional random sample survey of the general population that was done in Zambia and South Africa as part of the HPTN 071 (PopART) study.^4^ That study was an ongoing cluster-randomised trial measuring the effect of a combination prevention intervention on HIV incidence at population level, measured in a population cohort of randomly sampled adults who are being followed up for 36 months. Full details of the study have been published elsewhere.^4^ The trial has been implemented in 21 study communities: nine in the Cape Metro District and Cape Winelands District of the Western Cape Province of South Africa and 12 in Zambia, spread across four provinces and six districts (appendix p 2).

The data used in this paper were taken from the baseline survey of the population cohort done between Nov 28, 2013, and March 31, 2015, and the laboratory-confirmed HIV status of all participants. In each of the 21 trial communities, a random sample of households was selected and visited by field staff who enumerated all adults aged 18–44 years. From this list, one adult from each household was randomly selected and provided informed consent to participate in the population cohort. Next, the entire population cohort survey was administered in the respondent's preferred language by trained field workers. The HRQoL questions were embedded as a section in the population cohort survey. From each respondent, detailed information was gathered about HIV testing, self-reported HIV status, sociodemographics, health, and economic and behavioural aspects. Respondents self-reported their HIV status. If they self-reported being HIV-positive, they were asked whether they were in HIV care, and whether and for how long they had been on ART. After completion of the survey, a research nurse offered all respondents an on-the-spot HIV rapid test with pretest and post-test counselling. HIV status was confirmed by testing of blood samples drawn from consenting participants (appendix p 3).

HRQoL information was gathered in South Africa with the certified translation of the EuroQol five dimensions, five levels questionnaire (EQ-5D-5L).^5^ Since no certified translation of the EQ-5D-5L was available for Zambia, the study team translated the questionnaire into regional Zambian dialects. The EQ-5D-5L measures HRQoL in five separate domains (mobility, self-care, ability to do daily activities, pain, and anxiety or depression) and each domain is measured with five levels (no problems, slight, moderate, severe, or unable to; appendix pp 3–4). Because the questions are not disease specific, the measured HRQoL of HIV-positive and HIV-negative people can be directly compared—an important feature for this study. EQ-5D has been used previously to study HRQoL in the general population and in people living with HIV in LMICs and high-income countries,^6^, ^7^, ^8^ and it is an appropriate generic tool for measuring HRQoL in patients with HIV/AIDS.^9^

A full ethics review of the trial protocol was done by the ethics committees of the University of Zambia, Stellenbosch University, the London School of Hygiene & Tropical Medicine, Imperial College London, and the US Centers for Disease Control and Prevention.

### Statistical analysis

We used multivariate β regression models to evaluate the effect of HIV status and ART on HRQoL scores. We selected complementary log–log link functions over logit, probit, and log–log alternatives on the basis of the model that minimised Bayesian information criterion.^10^ Two defining properties of the HRQoL utility score guided selection of the regression model. First, it has truncated support (ranging between 0 and 1). Second, as in the case of other studies,^7^ it was negatively skewed with a spike at the upper end of the scale. Such models have been widely applied in analysing variables that are constrained between 0 and 1 and are either positively or negatively skewed.^11^, ^12^, ^13^

β regressions are more robust than other commonly used approaches in estimating covariate effects on HRQoL.^14^ We used the betareg routine in Stata (version 14). Results are presented as marginal effects, whereby a negative effect represents the magnitude of reduction in the score. With HIV-negative individuals as the base case, the model included people with HIV in five categories: HIV positive and unaware of status (those reporting being negative or unaware of their status, but confirmed as positive from the laboratory tests); HIV positive and aware of status, but not in HIV care; HIV positive and in HIV care, but not yet on ART; HIV positive and on ART initiated within the last 5 years; and HIV-positive people who initiated ART 5 or more years previously. The model included the adjustment variables age, sex, education, religion, ethnic group, herpes simplex virus type 2 status, and use of recreational drugs. We also included trial cluster dummy variables to capture community-level unobservable differences. We ran models separately for Zambia and South Africa. The appendix provides results for alternative specifications.

We analysed the five domains of HRQoL to determine which domains contributed to the observed effects on HRQoL. We used seemingly unrelated ordered probit regressions to take into account that an individual's responses in each of the five domains might be correlated. For example, individuals reporting problems with mobility might also be more likely to report problems completing daily activities. This approach is a generalisation of the standard ordered probit regression model allowing for the error terms of each individual's responses in the five domains to be correlated. In this case, we had five ordered probit equations (one for each domain) with error terms correlated across the five models. Negative marginal effects show the reduction in the probability of reporting no problems in the specific domain of health. We did the analysis with the *cmp* routine in Stata (version 14).

We used the results of the HRQoL score regressions to quantify the average quality-adjusted life-years (QALYs) that might be gained from treatment. For example, assuming each untreated HIV-positive individual has 10 remaining years of life, irrespective of current age or disease stage, and those on ART have remaining years of life according to life tables by country, age, and sex, we can combine the remaining years of life with the predicted HRQoL scores for each country to generate the value of remaining years of life, taking into account the extension of life and HRQoL.

### Role of the funding source

The funders of the study had no role in the study design, data collection, data analysis, data interpretation, or writing of the report. RT and KH had full access to the data in the study. RT had final responsibility for the decision to submit for publication.

## Results

The full sample included responses from 19 750 (83%) of 23 676 randomly selected individuals in Zambia and 18 941 (88%) of 21 568 randomly selected individuals in South Africa. HIV status from laboratory-tested blood samples was available for 19 330 (98%) participants in Zambia and 18 004 (95%) participants in South Africa. 4128 (21%) of these 19 330 respondents in Zambia and 4012 (22%) of 18 004 respondents in South Africa had laboratory-confirmed HIV. 19 637 (99%) participants in Zambia and 18 429 (97%) participants in South Africa had complete EQ-5D-5L information.

Prevalence of HIV in the trial communities was similar in both countries (table 1). A large proportion of HIV-positive participants were unaware of their status (table 1). Of HIV-positive participants aware of their HIV status, more reported being on ART in Zambia than in South Africa (table 1). Both countries had lower proportions of male respondents than female respondents (table 1). The unadjusted results show that HIV-positive people in Zambia reported lower levels of HRQoL than HIV-negative people, particularly in the domain of pain, which had a 4 percentage-point difference between the two groups (table 2). Except for a significant difference in the domain of anxiety or depression, there was no difference in HRQoL between HIV-positive and HIV-negative individuals in South Africa. Mean HRQoL score in HIV-positive and HIV-negative people was 0·88 in Zambia and 0·89 in South Africa (table 2).

**Table 1:** Sample demographics

		**Zambia (n=19 750)**	**South Africa (n=18 941)**
Age (years)		27 (7·2)	29 (7·4)
HRQoL score		0·88 (0·1)	0·89 (0·03)
Sex			
	Male	5428/19 733 (28%)	5816/18 612 (31%)
	Female	14305/19 733 (73%)	12796/18 612 (69%)
Ethnic group			
	1	5827/19 750 (30%); Bemba	12 048/18 941 (64%); Xhosa
	2	2453/19 750 (12%); Tonga	4803/18 941 (25%); multiracial
	3	1547/19 750 (8%); Lozi	526/18 941 (3%); Afrikaner
	4	1404/19 750 (7%); Chewa	1564/18 941 (8%); other
	5	8519/19 750 (43%); other*	··
Christian		19 479/19 680 (99%)	15 140/18 270 (83%)
Educational level			
	School education less than grade 8 (primary school)	5544/19 668 (28%)	1472/18 466 (8%)
	School education between grades 8 and 12 (secondary school)	12 808/19 668 (65%)	15 947/18 466 (86%)
	College, university, or other higher education	1316/19 668 (7%)	1047/18 466 (6%)
HSV-2-positive		8117/19 234 (42%)	8870/17 857 (50%)
Use recreational drugs		480/19 629 (2%)	689/18 432 (4%)
Alcohol consumption†		970/19 732 (5%)	1145/18 770 (6%)
HIV-negative		15 202/19 330 (79%)	13 992/18 004 (79%)
HIV-positive‡		4128/19 330 (21%)	4012/18 004 (22%)
	HIV-positive, unaware of status	1768/4128 (43%)	2026/4012 (50%)
	HIV-positive, aware of status, not in HIV care§	487/4128 (12%)	350/4012 (9%)
	HIV-positive, in HIV care, not yet on antiretroviral therapy§	177/4128 (4%)	173/4012 (4%)
	HIV-positive, on antiretroviral therapy§	1585/4128 (38%)	1236/4012 (31%)
	Status unknown	111/4128 (3%)	227/4012 (6%)

Data are mean (SD), n (%), or n/N (%). HRQoL=health-related quality of life. HSV-2=herpes simplex virus type 2.

* All other ethnic groups varied between 0·03% and 6·69%.

† Participant drinks five or more drinks of alcohol two or more times a week.

‡ Numbers based on laboratory confirmed test results.

§ Numbers based on responses by those self-reporting being HIV-positive in the survey.

**Table 2:** Five health domain classifications for Zambia and South Africa

		**Zambia**			**South Africa**		
		HIV-negative (n=15 145)*	HIV-positive (n=4102)*	p value for difference†	HIV-negative (n=13 648)*	HIV-positive (n=3898)*	p value for difference†
Mobility		··	··	p<0·0001	··	··	p=0·25
	No problems walking around	14 727 (97%)	3905 (95%)	··	13 435 (98%)	3847 (99%)	··
	Slight or moderate problems walking around	389 (3%)	169 (4%)	··	199 (2%)	48 (1%)	··
	Severe problems or unable to walk around	29 (<1%)	28 (<1%)	··	14 (<1%)	3 (<1%)	··
Self-care		··	··	p<0·0001	··	··	p=0·18
	No problems washing and dressing myself	14 810 (98%)	3932 (96%)	··	13 407 (98%)	3842 (99%)	··
	Slight or moderate problems washing and dressing myself	320 (2%)	156 (4%)	··	235 (2%)	53 (1%)	··
	Severe problems or unable to wash or dress myself	15 (<1%)	14 (<1%)	··	6 (<1%)	3 (<1%)	··
Daily activities		··	··	p<0·0001	··	··	p=0·38
	No problems doing my usual activities	14 608 (97%)	3860 (94%)		13 337 (98%)	3801 (98%)	··
	Slight or moderate problems doing my usual activities	516 (3%)	226 (6%)	··	301 (2%)	91 (2%)	··
	Severe problems or unable to do my usual activities	21 (<1%)	16 (<1%)	··	10 (<1%)	6 (<1%)	··
Pain		··	··	p<0·0001	··	··	p=0·12
	No pain or discomfort	13 201 (87%)	3425 (83%)	··	13 068 (96%)	3710 (95%)	··
	Slight or moderate pain or discomfort	1850 (12%)	640 (16%)	··	568 (4%)	181 (5%)	··
	Severe or extreme pain or discomfort	94 (<1%)	37 (1%)	··	12 (<1%)	7 (<1%)	··
Anxiety or depression		··	··	p<0·0001	··	··	p=0·02
	Not anxious or depressed	13 873 (92%)	3642 (89%)	··	13 069 (96%)	3699 (95%)	··
	Slightly or moderately anxious or depressed	1186 (8%)	424 (10%)	··	540 (4%)	188 (5%)	··
	Anxious or depressed	86 (<1%)	36 (1%)	··	39 (<1%)	11 (<1%)	··
HRQoL score		0·88 (0·04)	0·88 (0·06)		0·89 (0·3)	0·89 (0·4)	

Data are n (%), n/N (%), or mean (SD), unless otherwise stated. HRQoL=health-related quality of life.

* Numbers based on complete responses to the five dimensions of HRQoL and laboratory-confirmed HIV status.

† p value (Wilcoxon rank-sum test) for the difference between HIV-negative and HIV-positive groups.

Regression results show that, in Zambia, individuals who initiated ART less than 5 years previously reported significantly lower HRQoL scores than HIV-negative individuals (table 3). However, the difference is small and unlikely to be clinically meaningful. We recorded no additional differences in HRQoL between HIV-negative and HIV-positive individuals (table 3). Results for South Africa show that HRQoL did not differ between HIV-positive individuals on ART and HIV-negative individuals (table 3). Compared with HIV-negative individuals, small reductions in HRQoL were reported by HIV-positive individuals who were aware of their status but not enrolled in HIV care and those in HIV-care but not yet on ART (table 3). Although significant, these magnitudes are again unlikely to represent meaningful reductions (table 3).

**Table 3:** Multivariable analysis of factors associated with health-related quality of life

	**Zambia (18 910 observations)**	**South Africa (16 805 observations)**
HIV-negative (base)	1 (ref)	1 (ref)
HIV-positive, unaware of status	−0·001 (−0·003 to 0·001); p=0·216	0·001 (−0·001 to 0·001); p=0·997
HIV-positive, aware of status, not in care	−0·002 (−0·01 to 0·001); p=0·223	−0·004 (−0·01 to −0·001); p=0·010
HIV-positive, in care, never taken ART	0·001 (−0·01 to 0·07); p=0·695	−0·008 (−0·01 to −0·004); p=0·0001
HIV-positive, initiated ART less than 5 years ago	−0·006 (−0·008 to −0·003); p<0·0001	−0·001 (−0·003 to 0·000); p=0·140
HIV-positive, initiated ART 5 years or more ago	−0·002 (−0·01 to 0·001); p=0·219	0·000 (−0·002 to 0·003); p=0·939
Age 18–25 years (base)	1 (ref)	1 (ref)
Age 25–34 years	−0·003 (−0·004 to −0·001); p<0·0001	0·00 (0·001 to 0·001); p=0·513
Age >35 years	−0·01 (−0·009 to −0·006); p<0·0001	−0·002 (−0·003 to −0·001); p=0·0002
Women (base)	1 (ref)	1 (ref)
Men	0·001 (0·000 to 0·002); p=0·151	0·001 (0·001 to 0·002); p=0·001
Bemba (base Zambia), Xhosa (base South Africa)	1 (ref)	1 (ref)
Tonga (Zambia), multiracial (South Africa)	0 (−0·002 to 0·002); p=0·827	0 (−0·001 to 0·001); p=0·0991
Lozi (Zambia), Afrikaner (South Africa)	0·002 (−0·001 to 0·004); p=0·149	−0·001 (−0·004 to 0·002); p=0·0446
Chewa (Zambia)	0 (−0·002 to 0·002); p=0·901	··
Other	−0·001 (−0·002 to 0·001); p=0·370	0 (−0·001 to 0·002); p=0·0618
Other religion (base)	1 (ref)	1 (ref)
Christian	0·001 (−0·004 to 0·006); p=0·727	0·001 (0·000 to 0·002); p=0·037
School education less than grade 8 (primary school, base)	··	··
School education between grade 8 and 12 (secondary school)	0·002 (0·000 to 0·003); p=0·013	0·003 (0·002 to 0·01); p<0·0001
College, university, or other higher education	0·002 (−0·001 to 0·004); p=0·112	0·004 (0·002 to 0·006); p=0·0007
HSV-2-negative (base)	1 (ref)	1 (ref)
HSV-2-positive	−0·001 (−0·002 to 0·000); p=0·088	0·001 (−0·000 to 0·002); p=0·102
Does not use recreational drugs (base)	1 (ref)	1 (ref)
Uses recreational drugs	−0·01 (−0·01 to −0·002); p=0·0009	−0·002 (−0·004 to 0·000); p=0·067
Community fixed effects	Yes	Yes

Data are change in mean health-related quality of life score (95% CI), unless otherwise stated. For all factor variables, each category is compared with the base category. ART=antiretroviral treatment. HSV-2=herpes simplex virus type 2.

When we analysed the five domains of HRQoL, results for Zambia showed that HIV-positive individuals who had initiated ART less than 5 years previously were less likely than HIV-negative individuals to report no problems across all five domains (table 4). In both countries, HIV-positive individuals on ART for at least 5 years had a similar HRQoL to HIV-negative individuals across all five domains (table 4). In South Africa, individuals in HIV care who had never taken ART were less likely than HIV-negative individuals to report no problems with mobility, self-care, and daily activities (table 4). In both countries, individuals aware of their HIV-positive status but not in HIV care were significantly less likely to report no anxiety or depression than were HIV-negative individuals (table 4).

**Table 4:** Multivariable analysis of dimensions of health-related quality of life in Zambia and South Africa

	**Zambia (n=18 964 observations)**					**South Africa (n=16 886 observations)**				
	Mobility	Self-care	Daily activities	Pain	Anxiety or depression	Mobility	Self-care	Daily activities	Pain	Anxiety
HIV-positive, unaware of status	−0·01 (−0·02 to 0·00); p=0·102	−0·01 (−0·01 to 0·00); p=0·180	−0·003 (−0·01 to 0·01); p=0·508	0·001 (−0·02 to 0·02); p=0·957	−0·004 (−0·02 to 0·01); p=0·604	0·001 (−0·00 to 0·01); p=0·820	0·001 (−0·00 to 0·01); p=0·614	0·001 (−0·01 to 0·01); p=0·797	0·01 (−0·00 to 0·02); p=053	0·01 (−0·00 to 0·01); p=0·165
Aware of HIV-positive status, not in care	0·001 (−0·01 to 0·01); p=0·909	0·003 (−0·01 to 0·01); p=0·612	−0·011 (−0·03 to 0·01); p=0·188	−0·024 (−0·06 to 0·01); p=0·121	−0·03 (−0·06 to −0·002); p=0·037	0 (−0·01 to 0·01); p=0·921	−0·01 (−0·03 to 0·00); p=0·127	−0·02 (−0·04 to 0·003); p=0·068	−0·015 (−0·04 to 0·01); p=0·151	−0·03 (−0·06 to −0·005); p=0·016
Aware of HIV-positive status, in care, never taken ART	−0·004 (−0·03 to 0·02); p=0·719	−0·03 (−0·06 to −0·00); p=0·033	−0·02 (−0·05 to 0·01); p=0·274	0·03 (−0·01 to 0·07); p=0·170	0·02 (−0·02 to 0·05); p=0·345	−0·04 (−0·07 to −0·01); p=0·015	−0·03 (−0·05 to −0·003); p=0·034	−0·06^†^ (−0·10 to −0·02); p=0·002	−0·03 (−0·07 to 0·003); p=0·070	−0·02 (−0·05 to 0·01); p=0·204
Initiated ART less than 5 years ago	−0·02 (−0·03 to −0·01); p=0·002	−0·02 (−0·03 to −0·01); p=0·002	−0·02 (−0·03 to −0·01); p=0·004	−0·04 (−0·06 to −0·01); p=0·002	−0·03 (−0·05 to −0·01); p=0·001	−0·01 (−0·02 to 0·001); p=0·080	−0·01 (−0·02 to 0·003); p=0·173	−0·02 (−0·03 to −0·00); p=0·018	−0·01 (−0·03 to 0·002); p=0·073	−0·02 (−0·03 to 0·002); p=0·051
Initiated ART at least 5 years ago	−0·01 (−0·03 to 0·00); p=0·125	−0·002 (−0·01 to 0·01); p=0·697	−0·015 (−0·03 to 0·00); p=0·085	−0·01 (−0·04 to 0·02); p=0·503	−0·01 (−0·03 to 0·01); p=0·438	−0·002 (−0·01 to 0·01); p=0·667	0·002 (−0·01 to 0·01); p=0·749	−0·01 (−0·02 to 0·01); p=0·387	−0·002 (−0·02 to 0·02); p=0·766	0·01 (−0·01 to 0·02); p=0·471

Data are marginal effects (95% CI). HIV-negative is the base category. A negative marginal effect shows the reduction in probability of reporting “no problems”. Models include the covariates age, gender, education, ethnic group, religion, uses recreational drugs, and herpes simplex virus type 2 status. ART=antiretroviral therapy.

We estimate that, on average, each HIV-positive individual on ART would gain 26·24 QALYs in South Africa and 26·20 QALYs in Zambia, compared with an untreated individual. If we project these data to the UNAIDS 2016 estimates of 3·64 million individuals not yet on ART in South Africa, treating 90% of these individuals would equate to a gain of roughly 86 million QALYs as a direct benefit. Similar estimates for Zambia would mean 10·4 million QALYs could be gained from reaching 90% of the 0·44 million HIV-positive individuals not yet on ART.

## Discussion

To our knowledge, this is the first and largest study to evaluate the differences in HRQoL between HIV-positive and HIV-negative individuals since the expansion of ART in LMICs with high HIV burden. Unlike most previous studies, which compared the HRQoL of HIV patients at clinics (who are often at advanced disease stages) with the HRQoL of the general population, this study is the first to evaluate HRQoL by awareness of infection and ART status in a random sample from the general population, using laboratory-confirmed HIV status. We estimated several multivariable models with different categorisations of HIV status. We did analyses separately for Zambia and South Africa. Although a multicountry analysis provides valuable added insight, the two countries have very different population and health-system characteristics; therefore, we refrained from a direct comparison of results between countries.

38% of HIV-positive individuals in Zambia and 31% in South Africa were receiving ART, and receipt of treatment raised their HRQoL to that of HIV-negative individuals. The only exception was individuals in Zambia who had initiated ART less than 5 years previously, who reported a lower HRQoL score than HIV-negative individuals; however, the difference was very small. Roughly 4% of HIV-positive people in both countries were in care and had not started ART. In South Africa, these individuals had lower HRQoL than HIV-negative individuals. This finding was due to the dimensions of mobility, self-care, and problems in doing daily activities, but differences in scores were small when compared with HIV-negative people. 12% of HIV-positive people in Zambia and 9% of those in South Africa were aware of their status but not linked to care. In both countries, these individuals were more likely to report being anxious or depressed than people without HIV. A high proportion of HIV-positive individuals were unaware of their status (43% in Zambia, 50% in South Africa). In both countries, these individuals reported the same HRQoL as HIV-negative individuals, possibly representing the asymptomatic nature of HIV infection in its earlier stages.

Modelling estimates for KwaZulu-Natal suggest that it would take an average of 4·9 years for 50% of HIV seroconverters to be linked to care.^15^ Our findings support the observation that, at any one time, most HIV-positive people do not receive care and are not even aware of their status, but report good health. Overall, our estimates of differences are small and possibly not clinically relevant at the individual level. However, when scaled up to population level, they constitute a substantial loss in QALYs. Our calculations suggest that nearly 100 million QALYs could be gained across the two countries if 90% of currently untreated individuals are on ART, but most of these gains are due to extension in length of life. Other research has shown that early mortality rates among adults accessing ART are high in the first year of ART in sub-Saharan Africa,^16^ and that many people enter care at an advanced stage of disease and with clinically significant comorbidities.^17^ Our findings call for strategies to avoid losses in HRQoL that occur before individuals receive ART, by aiming at early diagnosis, timely initiation of ART, and improvement of adherence. Delays in health-systems initiation of ART must be minimised, especially in patients who present with advanced immunodeficiency.

Previous studies from high-income countries^6^, ^18^, ^19^, ^20^ and LMICs^21^, ^22^ found that average HRQoL of HIV-positive individuals was overall lower than that of HIV-negative individuals. However, evidence is contradictory as to whether HIV-positive individuals with asymptomatic disease or viral suppression have the same^20^ or lower^6^ HRQoL than HIV-negative people. We found smaller magnitudes of differences in HRQoL, by contrast with previous studies that compared clinical cohorts with the general population. In our sample from the general population, almost 60% of HIV-positive people belonged to one of two groups—either unaware of their status and potentially still in good health, or stable on ART for over 5 years and therefore also in relatively good health. Therefore, comparison of our findings with previous studies is problematic. Additionally, all but one of these studies was done before access to testing and treatment was accelerated. Most previous studies also sampled patients enrolled in HIV care, who were likely to be at a more advanced stage of disease and not representative of the overall population of people living with HIV.^18^, ^20^, ^21^, ^22^

The main strengths of this study are that data were gathered recently, covered a large sample of the general population, comprised both HIV-negative and HIV-positive people from two countries, and enabled adjustment for several confounders that were collected for both groups in the same way. This approach allowed us to provide a rare insight into the HRQoL of HIV-positive individuals at different stages of engagement with HIV care, including those who were not aware of their status or who were aware but not in HIV care. As the largest survey of HRQoL in these countries, our survey findings provide an important resource of quality-of-life estimates for future studies.

Our study has limitations. Blood samples from respondents were tested for their HIV status, but no information about disease stage was available. Therefore, we could not differentiate HRQoL by confirmed disease stage. However, evidence shows that in sub-Saharan Africa, mean CD4 cell count at ART initiation has remained at about 152 per μL in the past decade.^17^ The group of individuals on ART in our study is thus likely to have been in more advanced clinical stages of HIV at treatment initiation, with associated lower HRQoL. Our results suggest that, with ART, average HRQoL scores recover to levels in the general population, a finding corroborated by clinical studies.^3^ We relied on self-reports of ART initiation, which might have been affected by recall bias. Men were under-represented in the sample because the survey was done during the day and fewer men were available at home for interviews. This imbalance might have biased results if there were systematic differences in reported HRQoL between sexes. Results from previous studies have suggested that women might report lower HRQoL than men at similar disease stages, but these studies used a different instrument.^23^, ^24^ Although we adjusted for a large number of possible confounders, some could have been unobserved and could have affected results if they differed systematically by HIV status. We had to use the health state valuations for Zimbabwe because valuations were not available for South Africa or Zambia. Stigma has been shown to substantially affect mental health of HIV-positive individuals,^25^ but this influence could be captured by the anxiety or depression dimension of the EQ-5D-5L.

The unique design of our study allowed us to identify the success of ART in reducing inequalities between the HRQoL of HIV-infected individuals and the HIV-negative population. But our findings are also a call to step up efforts to extend these benefits to the millions of people not yet on ART. Improved access to ART is considered the main reason for the marked increase in overall life expectancy in sub-Saharan Africa over the last decade.^26^, ^27^, ^28^ Additionally, ART can reduce rates of sexual transmission of HIV,^29^ and substantial reductions in incidence, with associated savings in future treatment costs, have been predicted.^30^, ^31^, ^32^, ^33^, ^34^, ^35^ However, the beneficial effect of ART on the HRQoL of HIV-positive individuals is often not the focus of attention. This noteworthy and direct benefit of treatment could provide important additional support to international advocacy efforts for the UNAIDS Fast-Track targets. Policy makers should remember the purpose of medical treatment is to add years to life, and life to years.
