# Supplementary material for: Differences in health-related quality of life between HIV-positive and HIV-negative people in Zambia and South Africa: a cross-sectional baseline survey of the HPTN 071 (PopART) trial
Source: Lancet Glob Health. 2017 Sep 27;5(11):e1133–41. doi: 10.1016/S2214-109X(17)30367-4 (PMC5640509; doi:10.1016/S2214-109X(17)30367-4)
Supplement: Supplementary appendix [file mmc1.pdf]

THE LANCET
Global Health

Supplementary appendix

This appendix formed part of the original submission and has been peer reviewed. We post it as supplied by the authors.

Supplement to: Thomas R, Burger R, Harper A, et al, on behalf of the HPTN 071 (PopART) Study Team. Differences in health-related quality of life between HIV-positive and HIV-negative people in Zambia and South Africa: a cross-sectional baseline survey of the HPTN 071 (PopART) trial. *Lancet Glob Health* 2017; published online Sept 27. http://dx.doi.org/10.1016/S2214-109X(17)30367-4.

Supplementary information for Health-Related Quality-of-Life of people living with HIV in Zambia and South Africa: A comparison with HIV-negative people in the cross-sectional baseline survey of the HPTN 071 (PopART) Trial

Ranjeeta Thomas, Ronelle Burger, Abigail Harper, Sarah Kanema, Lawrence Mwenge, Nosivuyile Vanqa, Nomtha Mandla, Peter C Smith, Sian Floyd, Peter Bock, Helen Ayles, Nulda Beyers, Deborah Donnell, Sarah Fidler, Richard Hayes, Katharina Hauck on behalf of the HPTN 071(PopART) Study Team

S1. Supplementary information on characteristics of study communities

Country	Community number	Adult HIV prevalence (%) [1]	HIV-infected on ART (%) [2]	Population size [3]
Zambia	1	16	23	42,898
	2	13	29	33,297
	3	17	15	38,081
	4	19	30	60,222
	5	17	18	45,234
	6	19	32	34,623
	7	16	13	129,221
	8	15	22	166,251
	9	16	25	124,284
	10	25	24	31,629
	11	18	27	55,011
	12	16	38	41,615
South Africa	13	19	35	34,096
	14	19	35	21,386
	15	19	35	38,059
	16	15	37	72,544
	17	18	28	37,084
	18	14	36	44,821
	19	11	25	36,009
	20	11	25	82,953
	21	12	18	45,067

Notes:
Source: Information in the table replicated from Hayes et al [1]
The identities of the communities are concealed because the PopART trial is still ongoing.

[1]Estimated from ZAMSTAR 2010 TB/HIV prevalence survey, for all Zambian communities, with age standardisation to the age structure of prevalence survey participants and assuming 50% of the adult population are men. For Western Cape communities, source of HIV prevalence data varies by triplet. For Triplet 5, community 13 was included in the ZAMSTAR trial and the ZAMSTAR 2010 TB/HIV prevalence survey data are used, as for Zambia. HIV prevalence is then assumed to be the same in communities 14 and 15. For communities 16, 17, 19, 20, and 21, sub-district level data on antenatal clinic (ANC) prevalence were used, with the assumption that adult HIV prevalence is 80% of the ANC prevalence value. Community 18 was included in the ZAMSTAR trial and the ZAMSTAR 2010 TB/HIV prevalence survey data are used.

[2]Estimated from ZAMSTAR 2010 TB/HIV prevalence survey data, for all Zambian communities. The number of HIV-positive adults among prevalence survey participants was estimated, separately for men and women, as the age-standardised HIV prevalence multiplied by the number of survey participants. The proportion of HIV-positive individuals on ART was then calculated as (number self-reported on ART)/(estimated number of HIV-positive survey participants), and assuming that 50% of the adult population are men. For Western Cape communities, data were used from October 2012 on (a) the number of individuals aged >15 years old on ART – measured either at community or sub-district level, (b) population size among individuals >15 years old – measured using census data either at community or sub-district level, and (c) HIV prevalence estimates. The number of HIV-positive individuals aged >15 years old was estimated as HIV prevalence × community (or sub-district) population size. The proportion of HIV-positive individuals on ART was then calculated as (number of individuals >15 years old on ART)/(estimated number of HIV-positive individuals aged >15 years old).

[3]Population size – for Zambia, based on 2001 census data; for Western Cape, based on 2011 census data.

S2. Details on the sample and the HIV testing

Blood samples were analysed in-country using a single 4th generation assay (Architect HIV Ag/Ab Combo Assay, Abbott Diagnostics, Delkenheim Germany). Further testing was performed at the HIV Prevention Trials Network (HPTN) Laboratory Center (Baltimore, MD, USA). Samples that had reactive results in-country were tested with a second 4th generation assay (GS HIV Combo Assay, Bio-Rad Laboratories, Redmond, Wa). For quality assurance, 10% of the samples that had non-reactive results in-country were tested again using the Architect HIV Ag/Ab Combo Assay. Samples with discrepant/discordant test results were tested with additional assays to determine HIV status.

S3. Supplementary information on the EQ-5D-5L

Health-related quality-of-life (HRQoL) is a multi-dimensional measure of individuals perceived health across domains related to physical, mental, emotional, and social functioning. It attempts to measure the subjective impact that diseases have on quality of life. This study uses the EQ-5D-5L to calculate HRQoL. The EQ-5D-5L measures HRQoL on five separate domains (mobility, self-care, ability to do daily activities, pain/discomfort and anxiety/depression) and each domain is measured with five levels (no problems, slight, moderate, severe or unable to). Each respondent is asked to indicate his/her health state against the most appropriate statement in each of the 5 dimensions, and the answer is recorded by the interviewer. The respondents answer on each question results in a 1-digit number expressing the level selected for that dimension. The digit for each dimension can be combined in a 5-digit number describing the respondent's health state. For example, a person who has slight problems in walking about, no problems in washing and dressing, moderate problems in doing usual activities, extreme pain and discomfort and who is severely anxious or depressed has a health state of '21354'.

In the next step, the HRQoL weight is calculated for each respondent's health state. Each 5-digit EQ-5D health state is converted to a specific HRQoL weight by applying a so-called value set, a formula that attaches a value to each EQ-5D 5-digit number. The values are bound between 0 (worst health state, usually death) and 1 (perfect health). The value sets are a major feature of the EQ-5D instrument and they represent the country- and culture-specific preferences for each EQ-5D health state. Ideally, they are determined with an econometric analysis of a general population survey conducted in a specific country. If value sets are elicited with a robust economic study, they can be interpreted as the utilities that individuals derive from a specific health state, and the HRQoL score can be considered utility values. EQ-5D-5L can capture the potentially severe compound effects that HIV/AIDS has on the HRQoL of individuals.[2]

The previous version of the EQ-5D measured each domain across 3 levels (no problems, some problems, extreme problems), and value sets have been elicited for many countries. However, studies on value sets for the more recently developed EQ-5D-5L are under development in a number of countries; these studies will take time to complete and for results to be disseminated. In the interim, the EuroQol Group coordinated studies that administered both the 3-level and 5-level versions of the EQ-5D, in order to develop "crosswalks" between the EQ-5D-3L value sets and the new EQ-5D-5L descriptive system, resulting in crosswalk value sets for the EQ-5D-5L.[3] The Crosswalk value set for Zimbabwe was used in this study, because it is the only sub-Saharan African country for which these values are available. The construction of the EQ-5D makes it possible to capture the potentially severe compound effects that HIV/AIDS has on the HRQoL of individuals. But because questions are generic, i.e. not disease specific, the HRQoL weights of HIV infected and uninfected can be directly compared, which is an important feature for this study.

HRQoL weights or utility values can be used to calculate QALYs. The QALY is a measure of the value of health outcomes. It was developed as an attempt to combine length of life and the value of HRQoL into a single index number. One QALY equates to one year lived in perfect health, and is represented as 1 Year of Life × 1 utility value = 1 QALY. If an individual's health is less than perfect, QALYs are accrued at a rate of less than 1 per year. In order to determine the exact QALY value, the utility value associated with a given state of health needs to be determined, by using the method described above, and multiplied by the years lived in that state. For example, if the utility of a health state were associated with a utility value of 0.5, a year lived in this health state were equivalent to 0.5 QALYs (1 years × 0.5 utility value). QALYs are therefore expressed in terms of "years lived in perfect health": half a year lived in perfect health is equivalent to 0.5 QALYs (0.5 years × 1 utility value), the same as 1 year of life lived in a situation with utility 0.5 (e.g. bedridden) (1 year × 0.5 utility value). Please consult the website of the EUROQOL Group for further details on the EQ-5D-5L (http://www.euroqol.org/about-eq-5d.html)

EQ-5D-5L can capture the potentially severe compound effects that HIV/AIDS has on the HRQoL health of individuals.[2] Each combination of EQ-5D-5L health states can be converted to a single index value, the HRQoL score, when combined with valuations of the health states from general population samples . The EuroQol group provides health state valuations for a range of countries. The health state valuations for Zimbabwe was used in this study, because it is the only sub-Saharan African country for which these values are available. The calculated HRQoL scores range between 0 and 1, where 0 represents death and 1 perfect health. In combination with information on duration of life, HRQoL scores can be used to calculate quality-adjusted life years (QALYs). A fundamental assumption underlying measurement of health outcomes in economic evaluations is that the HRQoL scores have the properties of an interval scale where an increase from 0·2 to 0·4 has the same value as an increase from 0·6 to 0·8.[4]

S4: Supplementary results

In the table below we present results for the HRQoL beta regressions with two alternative specifications in addition to the models described in the main text. These models were sequentially estimated to evaluate the robustness of our results to the addition of explanatory variables and the expansion of our main variable of interest – HIV status. First, HIV status was categorised as a binary indicator representing HIV-positive or HIV-negative status adjusting for age and gender, we next expanded HIV status to the following categories – HIV-positive unaware of status (measured as those reporting being negative or unaware of their status but confirmed as positive from the blood samples), HIV-positive – aware of status but not in HIV-care, HIV-positive - in HIV-care but not yet ART, and in HIV-care on ART. Control variables also included education, religion, ethnic group, Herpes simplex virus type 2 (HSV2) status and the use of recreational drugs. The results for both countries remained unchanged when additional adjustment variables were included, namely wealth index, diagnosis of TB in the last 12 months, having a disability and being a heavy drinker (defined as five or more drinks at a time, 2 or more times per week).

Table: Multivariable analysis of factors associated with health-related quality-of-life in Zambia

	Zambia		South Africa	
	Change in mean HRQoL score[1]	Change in mean HRQoL score[1]	Change in mean HRQoL score[1]	Change in mean HRQoL score[1]
	Model 1	Model 2	Model 1	Model 2
HIV-negative (base)	---	---	---	---
HIV-positive, all	-0·003***		-0·001	
	[-0·004, -0·002]		[-0·002,0·000]	
HIV-positive, unaware of status		-0·001		0·001
		[-0·003,0·001]		[-0·001,0·001]
HIV-positive, aware of status, not in care		-0·002		-0·004**
		[-0·005,0·001]		[-0·01, -0·001]
HIV-positive, in care, never taken ART		-0·001		-0·01***
		[-0·006,0·004]		[-0·01, -0·004]
HIV-positive, on ART		-0·005***		-0·001
		[-0·01, -0·003]		[-0·002,0·001]
HIV-positive, initiated ART less than 5 years ago				
HIV-positive, initiated ART 5 years or more ago				
Age: 18- 25 years (Base)	----	----	----	----
Age: 25 years to 34 years	-0·003***	-0·003***	0·00	0·00
	[-0·004, -0·002]	[-0·004, -0·001]	[-0·001, 0·001]	[0·001, 0·001]
Age: 35 years and over	-0·01***	-0·01***	-0·002***	-0·002***
	[-0·010, -0·008]	[-0·009, -0·006]	[-0·003, -0·001]	[-0·003, -0·001]
Women (base)	---	---	---	---
Male	0·001	0·001	0·001	0·001***
	[-0·000,0·002]	[-0·000,0·002]	[0·000,0·001]	[0·001,0·002]
Ethnic group: Bemba (base Zambia)/ Xhosa (base- SA)	---	----	---	----
Tonga (Zambia) / Coloured (SA)		0		0
		[-0·002,0·002]		[-0·001,0·001]
Lozi (Zambia)/ Afrikaner (SA)		0·002		-0·001
		[-0·001,0·004]		[-0·004,0·002]
Chewa (Zambia)		0		
		[-0·002,0·002]		

Other		-0·001		0
		[-0·002,0·001]		[-0·001,0·002]
Other religion (base)			---	---
Christian		0.001		0·001**
		[-0.004,0.006]		[0·000,0·002]
School education less than grade 8 (primary school) (base)		----	---	---
School education between grade 8 to 12 (secondary school)		0.002**		0·003***
		[0.000,0.003]		[0·002,0·01]
College/University or other higher education		0·002		0·004***
		[-0·001,0·004]		[0·001,0·006]
HSV-2-negative (base)	---	---	---	---
HSV-2-positive		-0·001		0·001
		[-0·003, -0·000]		[-0·000,0·002]
Does not use recreational drugs (base)	---	---	---	---
Uses recreational drugs		-0·01**		-0·002
		[-0·006, -0·002]		[-0·004,0·000]
Community fixed effects	Yes	Yes	Yes	Yes
observations	19138	18910	17404	16805

References:

1. Hayes R, Ayles H, Beyers N, et al. HPTN 071 (PopART): rationale and design of a cluster-randomised trial of the population impact of an HIV combination prevention intervention including universal testing and treatment - a study protocol for a cluster randomised trial. *Trials* 2014; **15**: 57.
2. Wu AW, Hanson KA, Harding G, et al. Responsiveness of the MOS-HIV and EQ-5D in HIV-infected adults receiving antiretroviral therapies. *Health Qual Life Outcomes* 2013; **11**: 42.
3. van Hout B, Janssen MF, Feng YS, et al. Interim scoring for the EQ-5D-5L: mapping the EQ-5D-5L to EQ-5D-3L value sets. *Value Health* 2012; **15**(5): 708-15.
4. Drummond MF, Sculpher MJ, Claxton K, Stoddart GL, Torrance GW. Methods for the Economic Evaluation of Health Care Programmes. Oxford, United Kingdom: Oxford University Press, 2015.
